# Modulation of Gut Microbial Composition by *Lactobacillus delbrueckii* subsp. *lactis* CKDB001 Supplementation in a High-Fat-Diet-Induced Obese Mice

**DOI:** 10.3390/nu17132251

**Published:** 2025-07-07

**Authors:** Jaeryang Chu, Chae-Won No, Hyunchae Joung, Kyung Hwan Kim, Chang Hun Shin, Jisu Lee, Jung-Heun Ha

**Affiliations:** 1 Microbiome Research Laboratory, Chong Kun Dang Bio (CKDBiO) Research Institute, Ansan 15604, Republic of Korea; 2 Department of Food Science and Nutrition, Dankook University, Cheonan 31116, Republic of Korea; 3 Chong Kun Dang Bio (CKDBiO) Research Institute, Ansan 15604, Republic of Korea; 4 Research Center for Industrialization of Natural Neutralization, Dankook University, Yongin 16890, Republic of Korea

**Keywords:** *Lactobacillus delbrueckii* subsp. *lactis*, probiotics, gut microbiota, metabolic dysfunction, high-fat diet, in vivo model, PICRUSt2

## Abstract

**Background/Objectives:** *Lactobacillus delbrueckii* subsp. *lactis* CKDB001 (LL) has demonstrated anti-inflammatory, antioxidant, and lipid-regulatory effects in vitro and in vivo, including attenuation of hepatic steatosis and modulation of lipid metabolism. Given the known interactions between host metabolism and gut microbiota, these findings suggest a potential role for LL in modulating microbial composition under conditions of diet-induced obesity. This study aimed to investigate the microbiome-related effects of LL using an established murine model. To evaluate the effect of LL supplementation on gut microbial composition and predict microbial metabolic functions in mice with high-fat diet-induced obesity. **Methods:** Male C57BL/6J mice were fed a high-fat diet and administered LL orally for 12 weeks. Fecal samples were collected and analyzed using 16S rRNA gene sequencing. Microbial taxonomic profiles were assessed using linear discriminant analysis effect size, and functional predictions were performed using PICRUSt2. **Results:** LL supplementation significantly altered the gut microbiota by increasing the relative abundance of *Lactobacillus* and other commensal taxa while reducing the prevalence of pro-inflammatory genera such as *Alistipes* and *Bilophila*. Functional prediction analysis revealed a downregulation of lipopolysaccharide and ADP-L-glycero-β-D-manno-heptose biosynthesis pathways. Microbial functions associated with carbohydrate metabolism and short-chain fatty acid production were enriched in the LL-treated group. **Conclusions:** LL modulated gut microbial composition and suppressed pro-inflammatory microbial pathways while enhancing beneficial metabolic functions in high-fat diet-fed mice. These findings support the potential of LL as a safe and effective microbiota-targeted probiotic for managing obesity-related metabolic disorders.

## 1. Introduction

Probiotic microorganisms have gained increasing attention because probiotics can regulate gut microbial communities and support host metabolic and immune homeostasis [1,2]. The growing interest in microbiota-targeted interventions underscores the importance of identifying novel probiotic strains with specific health benefits [3,4,5]. Among these, various strains within the *Lactobacillus* genus have demonstrated health-promoting functions, such as enhancing intestinal integrity and modulating systemic physiology [6,7,8]. Beyond gut and metabolic health, probiotics have been investigated as adjunctive therapies for a range of chronic conditions. For example, recent clinical and experimental studies have shown that probiotic supplementation may alleviate symptoms of allergic rhinitis [9], improve glycemic control in adults with type 2 diabetes [10], and modulate immune responses relevant to the pathogenesis of psoriasis [11]. Building upon these perspectives, *Lactobacillus delbrueckii* supsp. *lactis* CKDB001 (LL) has recently emerged as a promising candidate with potential probiotic activity [12,13].

Although preliminary findings support its functionality, comprehensive evaluation across biological systems remains limited. A systematic investigation using both in vitro and in vivo models is essential to establish the physiological relevance and functional mechanisms of LL. Previous studies have elucidated the biological properties of LL. In vivo experimental trials have demonstrated its anti-inflammatory capacity, as evidenced by the suppression of tumor necrosis factor-α and interleukin-6 in immune-stimulated cells [12]. In addition, LL exhibited antioxidant activity and mitigated lipid accumulation in HepG2 hepatocytes by regulating key metabolic genes—specifically, upregulating SIRT1 and PPARα and downregulating CD36 and ELOVL6 [13]. These metabolic gene alterations suggest potential utility in managing metabolic dysfunction-associated steatotic liver disease. Furthermore, LL showed favorable safety characteristics, including the absence of hemolytic activity and lack of gelatinase, biogenic amines, or d-lactate production [13]. However, the limited translational capacity of cell-based models necessitates validation in whole-organism systems.

To extend these findings, a high-fat diet (HFD)-induced obesity mouse model was employed to assess the in vivo effects of LL [12]. Oral supplementation resulted in reduced body weight gain and prevented dyslipidemia [12]. Histological evaluation confirmed attenuation of hepatic steatosis and restoration of hepatic architecture [12]. Circulating inflammatory markers were significantly lowered, consistent with in vivo anti-inflammatory responses [12]. Importantly, no adverse clinical signs were observed throughout the study period, indicating the safety and efficacy of the strain in vivo [12]. Although these outcomes demonstrate the therapeutic potential of LL, the underlying role of gut microbiota modulation remains unexplored.

The gut microbiota plays a central role in maintaining metabolic and immune balance [14,15,16,17,18]. Disruption of microbial composition, referred to as dysbiosis, has been implicated in the pathogenesis of obesity [19,20], type 2 diabetes [21,22], and inflammatory bowel disease [23,24]. Probiotic interventions can restore microbial balance, enhance epithelial barrier function, reduce systemic inflammation, and improve metabolic outcomes [13,25,26,27]. Advances in 16S rRNA gene sequencing have enabled high-resolution profiling of microbial communities, facilitating the identification of taxonomic changes associated with therapeutic effects [28,29]. Therefore, evaluating microbiota shifts following probiotic supplementation provides crucial insights into host–microbe interactions and the mechanistic basis of probiotic action [30,31,32,33].

Despite the promising physiological improvements observed in previous studies, the impact of LL on gut microbiota composition remains unexplored. Given that many probiotic actions are mediated by microbial modulation, profiling LL-induced shifts is essential for elucidating its underlying mechanisms. The gut microbiota serves as a dynamic interface between diet and host metabolism, and changes in its composition, particularly under metabolic stress―such as HFD feeding―can substantially influence health outcomes. To date, no study has examined how LL alters the gut microbial landscape in the context of diet-induced metabolic dysfunction. Characterizing these microbial responses would not only clarify the functional role of LL but also help identify key taxa linked to metabolic improvements, thereby supporting targeted strategies for probiotic development and personalized interventions.

To address this knowledge gap, the present study investigated the impact of LL on gut microbial composition in an HFD-induced mouse model. The specific objectives were: (1) to evaluate the effect of LL supplementation on the gut microbiota using 16S rRNA gene sequencing; (2) to identify differentially abundant bacterial taxa associated with LL administration; and (3) to predict microbial metabolic functions using PICRUSt2 and assess their potential associations with metabolic and inflammatory improvements. These analyses aimed to elucidate the contribution of microbiota modulation to the probiotic effects of LL and to identify microbial signatures relevant to the management of metabolic diseases.

## 2. Materials and Methods

### 2.1. Preparation of LL

LL (KCTC 14149BP) was originally isolated from fermented milk. For culture preparation, LL was cultured in an optimized medium based on De Man, Rogosa, and Sharpe broth at 37 °C for 16–18 h, with the pH maintained between 5.5 and 6.0. Following cultivation, bacterial cells were harvested via centrifugation at 6000 rpm for 10 min (Hanil, Supra R12, Daejeon, Republic of Korea). The resulting cell pellet was lyophilized using a freeze-dryer (Cooling and Heating System; Lab-Mast 10, Seoul, Republic of Korea). The colony-forming units (CFU) per gram of lyophilized LL powder were quantified prior to in vivo administration.

### 2.2. Animals and Experimental Design

Forty-eight male C57BL/6J mice, 4 weeks old, were obtained from DooYeol Biotech (Seoul, Republic of Korea). Upon arrival, experimental mice were housed under controlled environmental conditions (22 ± 2 °C, 55 ± 5% humidity, and a 12-h light/dark cycle) and acclimated for one week. Following acclimation, mice were randomly assigned to four experimental groups (*n* = 12 per group), with three mice housed per cage, resulting in four cages per group. Experimental mice were fed either a standard chow diet or a high-fat diet (HFD) providing 60% of total energy from fat (Research Diets Inc., New Brunswick, NJ, USA) for 24 weeks. During the first 12 weeks, only the assigned diets were administered. For the following 12 weeks, treatment groups received a daily oral gavage of *Lactobacillus delbrueckii* subsp. *lactis* CKDB001 (LL; 1 × 10^9^ CFU suspended in 200 µL sterile phosphate-buffered saline [PBS]). The positive control group received a daily oral dose of resmetirom (3 mg/kg body weight; MedChemExpress, Beytelsbach, Germany), a thyroid hormone receptor-β agonist known to improve metabolic dysfunction. Control mice received an equivalent volume of sterile PBS without probiotics. Oral administration was selected to represent a potential clinical route for future preventive or therapeutic applications.

To minimize potential confounders, mice were randomly allocated to treatment groups, and cage positions were rotated weekly within the animal facility to reduce location-related environmental bias. The order of treatments and measurements was also randomized across groups to prevent order effects. All procedures, including dosing and sample collection, were performed at consistent times each day to minimize circadian variability. These measures were implemented to ensure uniform treatment conditions and reduce systematic bias. All animal procedures were approved by the Institutional Animal Care and Use Committee of Dankook University (No. DKU 2023-22-085) and conducted in accordance with institutional ethical guidelines. This study was conducted and reported in accordance with the ARRIVE guidelines 2.0 to ensure transparency and reproducibility in animal research [34].

### 2.3. 16S rRNA Amplicon Sequencing and Microbiota Analysis

At the end of the feeding period, fecal samples were collected aseptically, flash-frozen in liquid nitrogen, and stored at −80 °C until analysis. Microbial genomic DNA was extracted using the QIAamp^®^ DNA Stool Mini Kit (QIAGEN, Hilden, Germany) based on the instructions of the manufacturer. The V3–V4 hypervariable regions of the bacterial 16S rRNA gene were amplified using region-specific primers. Amplicons were purified and sequenced using an Illumina MiSeq platform (Illumina Inc., San Diego, CA, USA). Raw sequence data were processed using the QIIME2 pipeline, with quality filtering, sequence denoising, paired-end merging, chimera removal, and feature-table generation performed using the DADA2 plugin. Alpha (assessed using Chao1 and Shannon indices) and beta (visualized using principal coordinate analysis [PCoA] based on Bray-Curtis dissimilarity) diversities were calculated to evaluate within-sample and between-sample microbial variations, respectively. Taxonomic classification was performed using both the NCBI and SILVA reference databases. Species-level annotation was conducted using local BLASTN (ncbi-blast-2.10.0+) against the NCBI RefSeq 16S rRNA database (updated 11 February 2020), while representative sequences were classified using the SILVA v138 99% full-length 16S rRNA database. Microbial composition was subsequently analyzed at the phylum and genus levels. Linear discriminant analysis effect size (LEfSe) was used to identify differentially abundant taxa between the groups. Functional pathway prediction was performed using PICRUSt2 based on 16S rRNA profiles. All sequencing and bioinformatic analyses, including DNA extraction and library preparation, were conducted by 3BIGS Co., Ltd. (Hwaseong, Republic of Korea).

### 2.4. Statistical Analysis

Statistical analyses and data visualization were performed using GraphPad Prism software (GraphPad, version 8.3.1, San Diego, CA, USA) and R (version 4.3.0, R Core Team, R Foundation for Statistical Computing, Vienna, Austria). Prior to statistical analysis, data were assessed for normality and homogeneity of variance. Between-group comparisons were performed using Student’s *t*-test, whereas multiple-group comparisons were conducted using a one-way analysis of variance, followed by Dunnett’s multiple comparison test. Differences in beta diversity were assessed using Permutational Multivariate Analysis of variance (PERMANOVA). Associations between microbial taxa and predicted functional pathways were evaluated using Spearman’s rank correlation. A *p*-value < 0.05 was considered statistically significant.

## 3. Results

### 3.1. LL Supplementation Modulates Gut Microbial Composition at the Phylum and Genus Levels

**Figure 1:**
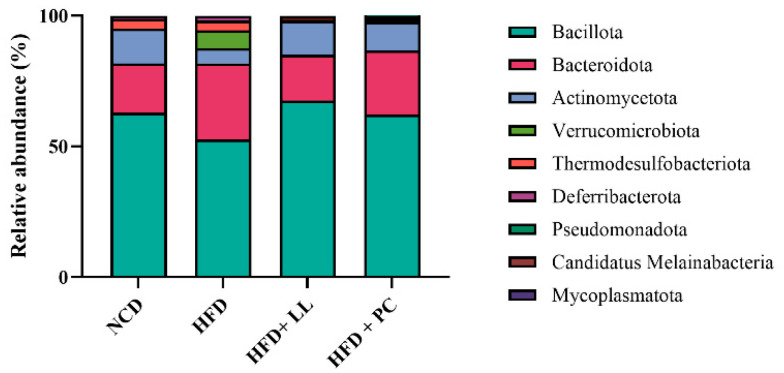
Bacterial phyla composition in the *L. lactis* CKDB001-supplemented mice. Abbreviations: NCD; Normal control diet supplemented group, HFD; High-fat diet supplemented group, HFD + LL; High-fat diet and *L. lactis* CKDB001 supplemented group, HFD + PC; High-fat diet and resmetirom-supplemented group.

**Figure 2:**
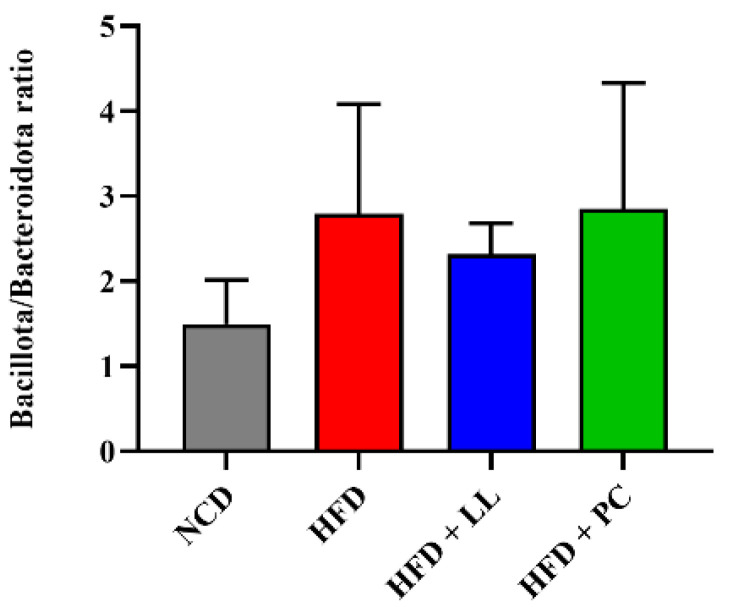
*Bacillota*/*Bacteroidota* ratio in *L. lactis* CKDB001-supplemented mice. Data are presented as mean ± standard deviation (SD). *n* = 12 per group. Abbreviations: NCD; Normal control diet supplemented group, HFD; High-fat diet supplemented group, HFD + LL; High-fat diet and *L. lactis* CKDB001 supplemented group, HFD +P C; High-fat diet with resmetirom-supplemented group.

**Figure 3:**
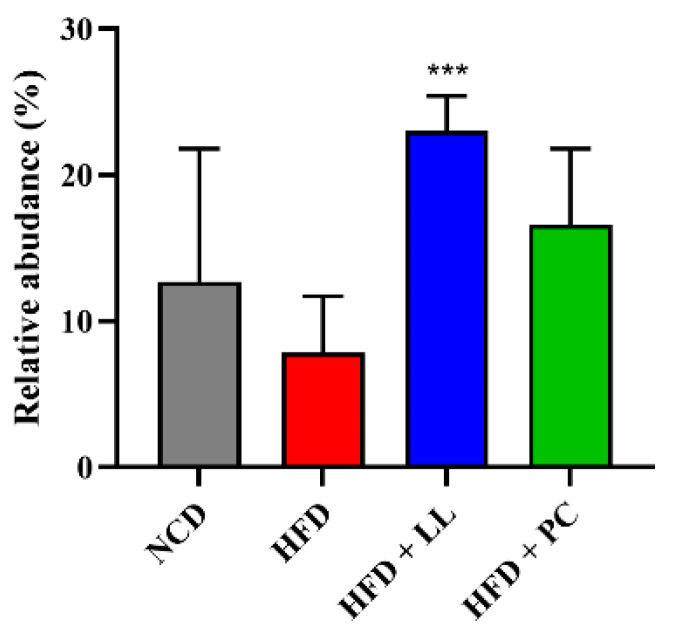
Relative abundance of *Lactobacillus* genus in *L. lactis* CKDB001-supplemented mice. Data are presented as mean ± standard deviation (SD). *n* = 12 per group. *p* values < 0.001 are indicated as “***”. Abbreviations: NCD; Normal control diet supplemented group, HFD; High-fat diet supplemented group, HFD + LL; High-fat diet and *L. lactis* CKDB001 supplemented group, HFD + PC; High-fat diet with resmetirom-supplemented group.

To evaluate the effects of LL on gut microbial communities, the relative abundance of bacteria was assessed at both the phylum and genus levels. Across all groups, *Bacillota* was the dominant phylum, followed by *Bacteroidota* and *Verrucomicrobiota*. In HFD-fed mice, the abundances of *Bacillota* and *Actinomycetota* were markedly reduced, whereas that of *Verrucomicrobiota* was elevated—hallmarks of HFD-induced dysbiosis (Figure 1). LL supplementation effectively attenuated these compositional disturbances, restoring *Bacillota* and *Actinomycetota* levels while suppressing the expansion of *Verrucomicrobiota* and *Bacteroidota*. Although the *Bacillota*-to-*Bacteroidota* ratio is commonly used as an indicator of dysbiosis in obesity, no significant group differences were observed between groups (Figure 2). LL administration significantly increased the relative abundance of *Lactobacillus*, which was substantially reduced in HFD-fed mice (Figure 3).

### 3.2. LL Enhances Microbial Alpha and Beta Diversity

**Figure 4:**
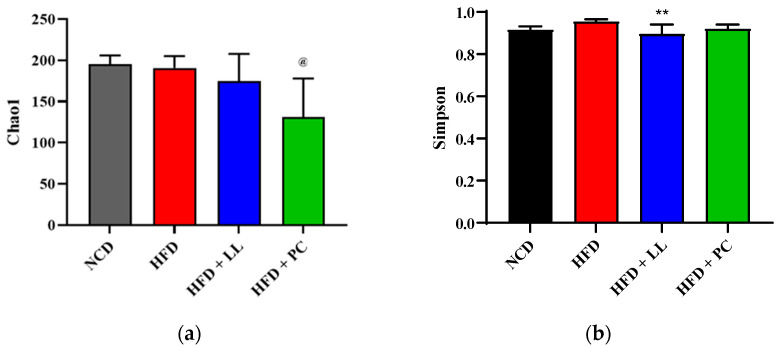
Alpha diversity index in *L. lactis* CKDB001-supplemented mice. *n* = 12 per group. (**a**) Chao 1 index, (**b**) Simpson index. Data are presented as mean ± standard deviation (SD). *p* values < 0.05 between HFD and HFD + PC are indicated by “@”, and *p* values < 0.01 between HFD and HFD + LL are indicated by “**”. Abbreviations: NCD; Normal chow diet supplemented group, HFD; High-fat diet supplemented group, HFD + LL; High-fat diet with *L. lactis* CKDB001 supplemented group, HFD + PC; High-fat diet with resmetirom-supplemented group.

To assess microbial richness and diversity, alpha diversity was measured using Chao1 and Simpson indices (Figure 4). Although the Chao1 index, which reflects species richness, showed no significant differences between the normal chow diet (NCD), HFD, and HFD + LL groups, the Simpson index, which emphasizes species evenness, revealed a significant increase in the LL group compared with the HFD controls. This suggests that LL contributed to a more balanced microbial community. In contrast, the PC group, treated with an FDA-approved anti-obesity drug, exhibited a reduction in alpha diversity, potentially reflecting unintended perturbation of microbial equilibrium.

**Figure 5:**
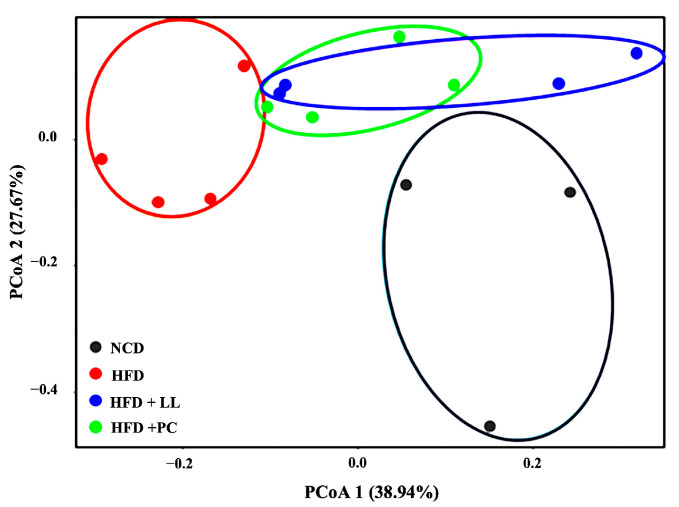
Beta diversity index based on Principal Coordinate Analysis (PCoA) in *L. lactis* CKDB001-supplemented mice. *n* = 3–4 per group. Abbreviations: NCD; Normal chow diet supplemented group, HFD; High-fat diet supplemented group, HFD + LL; High-fat diet with *L. lactis* CKDB001 supplemented group, HFD + PC; High-fat diet with resmetirom-supplemented group.

Beta diversity analysis using Bray–Curtis-based PCoA demonstrated a distinct separation between the NCD and HFD groups (PERMANOVA, R^2^ = 0.424, *p* = 0.029), confirming HFD-induced shifts in microbiota structure. Importantly, the LL group showed a clustering pattern clearly distinct from that of the HFD group (PERMANOVA, R^2^ = 0.368, *p* = 0.028), indicating that LL significantly restructured the gut microbial composition. A comparable but taxonomically distinct shift was also observed in the PC group (Figure 5).

### 3.3. LL Induces Taxonomic and Functional Shifts in Gut Microbiota

**Figure 6:**
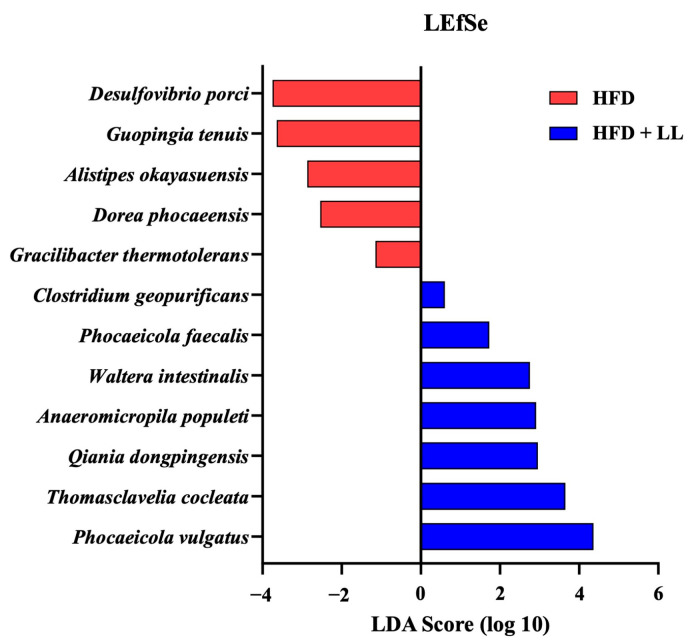
Distinctive signature of gut microbial species using LEfSe analysis in *L. lactis* CKDB001-supplemented mice. *n* = 12 per group. LDA scores showing significant differences between groups with alpha ≤ 0.01. Abbreviations: HFD; High-fat diet supplemented group, HFD + LL; High-fat diet with *L. lactis* CKDB001 supplemented group.

**Figure 7:**
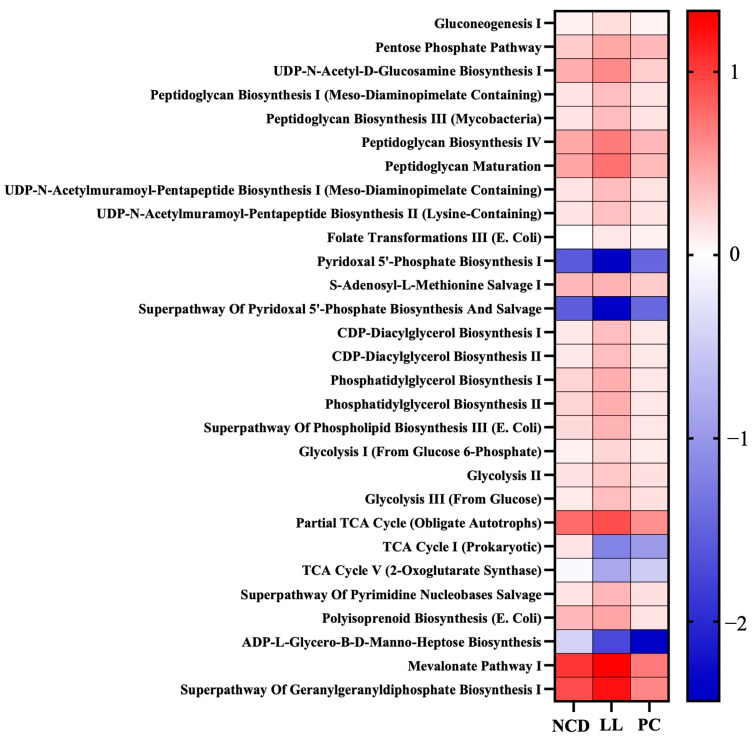
Functional pathway prediction using PICRUSt2 analysis in *L. lactis* CKDB001-supplemented mice. *n* = 12 per group. Differentiating pathways were identified using MaAsLin2 with a q-value of <0.05. Abbreviations: NCD; Normal chow diet supplemented group, LL; High-fat diet with *L. lactis* CKDB001 supplemented group, PC; High-fat diet with resmetirom-supplemented group.

To further characterize taxonomic and functional changes, LEfSe and PICRUSt2 analyses were performed (Figure 6 and Figure 7). LEfSe analysis identified several differentially enriched taxa in the LL group, including *Phocaeicola vulgatus*, whereas others, such as *Desulfovibrio porci*, were significantly depleted compared with the HFD group. Notably, the increased abundance of *P. vulgatus*—a species previously associated with improved hepatic function in metabolic liver disease—suggests a potential mechanistic link between LL-mediated microbial modulation and host metabolic benefits.

Functional prediction using PICRUSt2 revealed that LL supplementation was associated with a significant reduction in pyridoxal 5′-phosphate and ADP-L-glycero-β-D-manno-heptose biosynthesis pathways. The former has been implicated in the progression of nonalcoholic fatty liver disease (NAFLD), whereas the latter contributes to lipopolysaccharide (LPS) biosynthesis and related inflammatory signaling. These results suggest that LL may exert protective effects by downregulating microbial functions involved in metabolic dysfunction and systemic inflammation.

### 3.4. Correlation Between Key Microbes and Functional Pathways

**Figure 8:**
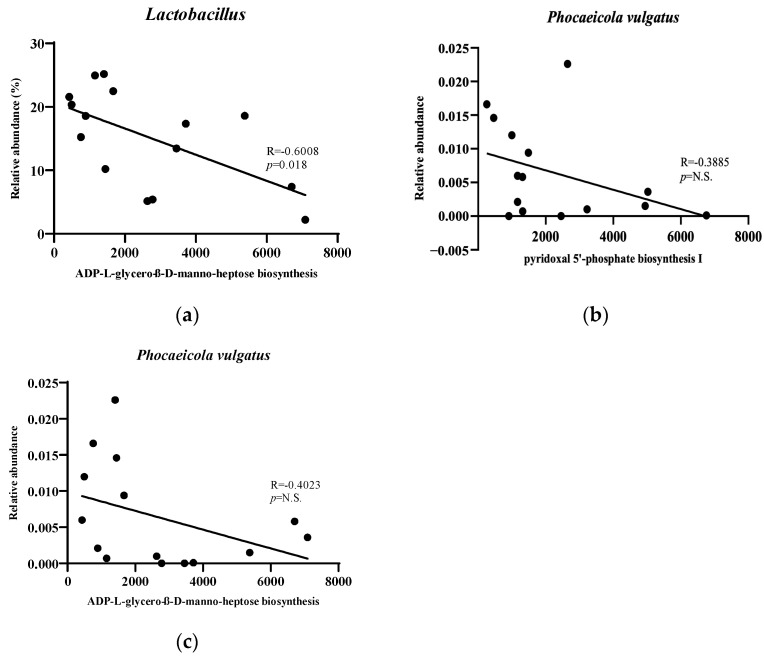
Correlation between gut microbial abundance and functional pathways. *n* = 12 per group. The *y*-axis represents the relative abundance of the gut microbiome based on metagenomic analysis results, and the *x*-axis represents the associated functional pathway, both of which were obtained through statistical significance tests. (**a**) *Lactobacillus* abundance and ADP-L-glycero-B-D-manno-heptose biosynthesis pathway; (**b**) *Phocaeicola vulgatus* abundance and pyridoxal 5′-phosphate biosynthesis I pathway; and (**c**) *Phocaeicola vulgatus* abundance and ADP-L-glycero-B-D-manno-heptose biosynthesis pathway.

**Figure 9:**
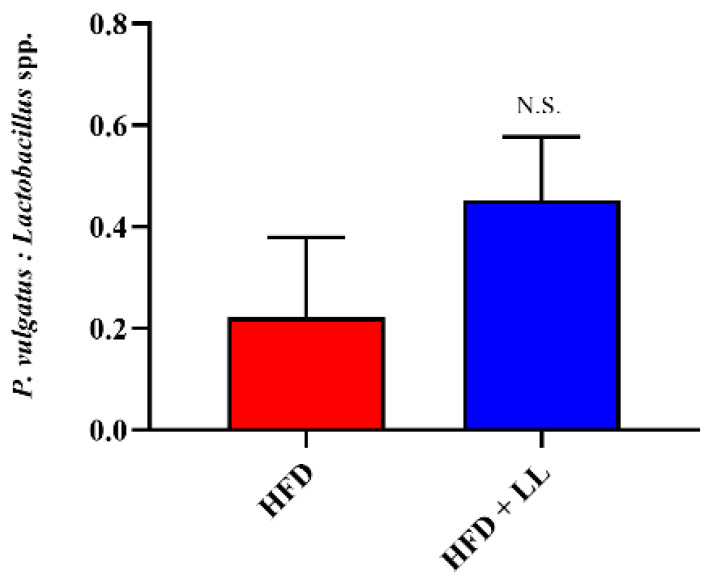
*Phocaeicola vulagtus*-*Lactobacillus* spp. genus ratio. *n* = 12 per group. Data are presented as mean ± standard deviation (SD). Abbreviations: HFD; High-fat diet supplemented group, HFD + LL; High-fat diet and *L. lactis* CKDB001 supplemented group, N.S.; Not significant.

Correlation analysis between specific microbial taxa and predicted functional pathways (Figure 8) revealed an inverse association between *Lactobacillus* abundance and the ADP-L-glycero-β-D-manno-heptose biosynthesis pathway, indicating a potential role in suppressing LPS production and reducing endotoxemia. Although *P. vulgatus* also showed negative correlations with pro-inflammatory pathways, these associations were not statistically significant. Interestingly, the LL-treated group exhibited a trend toward increased *P. vulgatus* to *Lactobacillus* spp. ratio compared with the NCD group (Figure 9), potentially reflecting a unique compositional shift induced by LL supplementation.

## 4. Discussion

This study comprehensively evaluated the functional properties of LL using animal- and microbiome-based approaches in the context of HFD-induced metabolic dysregulation. Exposure to LL suppressed lipid accumulation in HepG2 hepatocytes and modulated lipid metabolism-associated genes such as SIRT1, PPARα, CD36, and ELOVL6 [13], providing mechanistic insights for subsequent in vivo investigation. In an HFD-fed mouse model, LL supplementation reduced body weight gain and improved lipid profiles, including reductions in total cholesterol and triglycerides [12]. Histological analysis revealed decreased hepatic steatosis and improved structural integrity of the liver tissue [12]. Inflammatory cytokine levels in liver samples were reduced following LL treatment, suggesting anti-inflammatory activity [12]. The absence of clinical symptoms and histopathological abnormalities confirmed the physiological safety of LL. The convergence of cellular and animal data supports the relevance of LL as a functional agent in the management of metabolic disorders.

Microbiota analysis further demonstrated that LL effectively restored microbial balance disrupted by HFD feeding. The relative abundances of *Bacillota* and *Actinomycetota*, phyla commonly associated with metabolic health, were significantly reduced in HFD-fed mice but normalized after LL supplementation. Overgrowth of *Verrucomicrobiota*, previously linked to inflammatory and metabolic disturbances [35], was also suppressed by LL supplementation. Although the *Bacillota*-to-*Bacteroidota* ratio showed no significant changes, the relative abundance of *Lactobacillus* increased substantially in response to LL supplementation. Previous studies have shown that *Lactobacillus* species contribute to lipid regulation and immune modulation [12], consistent with current physiological observations. The Simpson index-based alpha diversity increased following LL supplementation, reflecting enhanced community evenness, whereas the Chao1-based richness remained stable. In contrast, the pharmaceutical comparator reduced overall alpha diversity, suggesting potential disruption of microbial homeostasis [36]. Bray-Curtis-based beta diversity analysis revealed distinct microbial clustering in the LL group relative to HFD controls, indicating a compositional shift toward a healthier gut ecosystem.

Functional prediction using PICRUSt2 revealed that LL supplementation downregulated microbiota-associated pathways linked to pyridoxal 5′-phosphate and ADP-L-glycero-beta-D-manno-heptose biosynthesis. These pathways have been implicated in the progression of NAFLD and LPS-driven inflammation, respectively [37,38]. The suppression of these microbial metabolic functions suggests that LL may contribute to hepatic protection and the reduction in inflammation through indirect microbial mechanisms. LEfSe analysis identified an enrichment of *Phocaeicola vulgatus* in LL-treated mice, a species previously associated with improved hepatic outcomes in metabolic disease models. An increased *P. vulgatus*-to-*Lactobacillus* ratio was observed in the LL group compared with the HFD group, which contrasts with prior observations linking a reduced ratio to obesity [39]. Conversely, taxa lacking metabolic benefits, such as *Duncaniella porci*, were significantly reduced. These findings emphasize that LL alters both microbial composition and functional capacity in a manner that supports host metabolic resilience. These microbial functional changes may contribute to reduced systemic inflammation and improved hepatic function by lowering endotoxin burden and supporting anti-inflammatory signaling. In particular, the suppression of LPS-related biosynthesis could attenuate immune activation, while the enhancement of SCFA-associated pathways may promote metabolic homeostasis [38,40].

The correlational analysis provided additional evidence for LL-mediated modulation of the gut microbiota. A strong negative association was observed between *Lactobacillus* abundance and the ADP-L-glycero-beta-D-mannoheptose biosynthetic pathway, indicating reduced LPS synthesis and a lower risk of endotoxin-related immune activation. This finding aligns with the observed decrease in inflammatory markers and supports the hypothesis that LL mitigates systemic inflammation by modulating microbiota-derived signals. Although *P. vulgatus* exhibited similar negative trends with inflammatory pathways, the trends were not statistically significant, potentially due to the variability in microbial composition or limited sample size. Overall, these patterns suggest that modulation of specific microbial taxa and pathways may underlie the metabolic and immunological benefits of LL supplementation. Functionally active probiotic strains, such as LL, may operate not only through direct interaction with host cells but also by reshaping microbial community structure and metabolic outputs [41,42]. However, it is important to acknowledge that the functional predictions generated by PICRUSt2 are inferred from the taxonomic composition of the microbial community. Consequently, the observed correlations between specific microbial taxa and predicted metabolic pathways do not represent independent biological measurements. These results should therefore be considered exploratory, offering hypothesis-generating insights rather than definitive evidence of mechanistic interactions [43].

Safety evaluations confirmed the probiotic suitability of LL for long-term use. No hemolytic activity, gelatinase production, biogenic amine formation, or D-lactate accumulation was detected in vitro [13]. During in vivo testing, no signs of clinical toxicity, behavioral abnormalities, or organ damage were observed [12]. Unlike pharmaceutical interventions, which often reduce microbial diversity, LL maintained or improved alpha diversity and community structure, reinforcing its compatibility with host microbial ecology. Sustaining microbial stability is critical for therapeutic strategies aimed at long-term metabolic regulation. These findings may have implications for clinical practice, particularly in the management of obesity-related metabolic disorders. Given the ability of LL to modulate gut microbial composition, suppress pro-inflammatory pathways, and enhance beneficial metabolic functions, LL may serve as a complementary strategy alongside existing dietary or pharmacological interventions. The observed microbiota shifts—including increased *Lactobacillus* abundance and reduced lipopolysaccharide biosynthesis pathways—align with microbial targets associated with improved metabolic outcomes in humans. Although further clinical validation is needed, the present results support the potential translational application of LL in microbiome-targeted approaches to chronic disease management.

This study had some limitations. Functional predictions relied on 16S rRNA gene sequences analyzed using PICRUSt2, which infers rather than directly measures metabolic pathways. Incorporation of shotgun metagenomics and untargeted metabolomics in future studies would enhance the accuracy of functional characterization and validate inferred pathways [44]. The use of a single mouse strain with diet-induced obesity may limit the generalizability of the findings, as host–microbe interactions can vary with genotype and environmental exposure. The use of germ-free or microbiota-depleted animal models could help isolate microbiota-specific effects. Although correlative analyses identified associations between microbial taxa and functions, causal relationships require confirmation through targeted microbial transplantation or depletion studies. Human-based trials are required to determine the clinical relevance of LL supplementation in patients with metabolic disorders. Despite these limitations, this study provides robust evidence that LL exerts microbiome-mediated benefits in the context of metabolic dysfunction and offers a foundation for further translational research.

## 5. Conclusions

LL exhibited multiple functional properties relevant to the prevention and management of obesity-associated metabolic dysfunction. In cellular studies, LL demonstrated antioxidant, anti-inflammatory, and antiadipogenic activities by regulating cytokine production and lipid metabolism-related genes. In a murine model of HFD-induced obesity, oral LL supplementation mitigated body weight gain, improved hepatic histopathology, and reduced systemic inflammatory response. Gut microbiota analysis indicated that LL restored microbial compositional balance and downregulated metabolic pathways associated with inflammation and dysregulation. Correlation analysis identified associations between key bacterial taxa, including *Lactobacillus* and *Parabacteroides vulgatus*, and the attenuation of pathogenic microbial functions. Safety assessments confirmed that LL is a well-tolerated strain that fulfills essential probiotic safety standards. These findings support the potential of LL as a microbiota-modulating probiotic candidate for therapeutic applications in metabolic disorders. Further validation using metagenomic profiling, mechanistic host-microbiota interaction models, and human clinical trials is necessary to confirm its translational applicability.

## Data Availability

The datasets used in this study are available from the corresponding author upon reasonable request.
